# Host-Driven Ubiquitination Events in Vector-Transmitted RNA Virus Infections as Options for Broad-Spectrum Therapeutic Intervention Strategies

**DOI:** 10.3390/v16111727

**Published:** 2024-10-31

**Authors:** Sanskruthi Sreepangi, Haseebullah Baha, Lorreta Aboagyewa Opoku, Naomi X. Jones, Maame Konadu, Farhang Alem, Michael D. Barrera, Aarthi Narayanan

**Affiliations:** 1 School of Systems Biology, College of Science, George Mason University, Fairfax, VA 22030, USA; ssreepan@gmu.edu (S.S.); hbaha@gmu.edu (H.B.); lopoku@gmu.edu (L.A.O.); njones16@gmu.edu (N.X.J.); mkonadu@gmu.edu (M.K.); mbarrer@gmu.edu (M.D.B.); 2 Institute of Biohealth Innovation, George Mason University, Fairfax, VA 22030, USA; falem@gmu.edu; 3 Department of Biology, College of Science, George Mason University, Fairfax, VA 22030, USA

**Keywords:** ubiquitination, vector-transmitted viruses, ubiquitin E3 ligases, host–virus interactions, alphaviruses, flaviviruses, bunyaviruses

## Abstract

Many vector-borne viruses are re-emerging as public health threats, yet our understanding of the virus–host interactions critical for productive infection remains limited. The ubiquitination of proteins, including host- and pathogen-derived proteins is a highly prominent and consistent post-translational modification that regulates protein function through signaling and degradation. Viral proteins are documented to hijack the host ubiquitination machinery to modulate multiple host processes including antiviral defense mechanisms. The engagement of the host ubiquitination machinery in the post-translational modification of viral proteins to support aspects of the viral life cycle including assembly and egress is also well documented. Exploring the role ubiquitination plays in the life cycle of vector-transmitted viral pathogens will increase the knowledge base pertinent to the impact of host-enabled ubiquitination of viral and host proteins and the consequences on viral pathogenesis. In this review, we explore E3 ligase-regulated ubiquitination pathways functioning as proviral and viral restriction factors in the context of acutely infectious, vector-transmitted viral pathogens and the potential for therapeutically targeting them for countermeasures development.

## 1. Introduction

Vector-borne viruses (VBVs) are an ongoing threat to public health, as they inflict a significant disease burden, morbidity, and often mortality on human and animal populations [1]. Left unchecked, VBVs have pandemic-causing potential as indicated by their global footprint. In particular, vector-borne RNA viruses are of utmost concern, as they have higher and faster mutation rates than DNA viruses [2]. Common vectors of VBVs include mosquitoes, ticks, flies, bats, fleas, and horseflies [1,3]. Current methods to control VBVs are mainly through vector control; however, this approach has not thus far resulted in the epidemiological control of associated disease spread, necessitating the development of therapeutic approaches to address disease. As several of these VBVs co-circulate in different parts of the globe in either common or unique vectors, therapeutic strategies that can achieve broad-spectrum outcomes will be highly desirable.

Ubiquitination is a post-translational protein modification process that regulates multiple cellular events including protein degradation, apoptosis, DNA damage, immune response, and cell survival [4]. Ubiquitination is a highly conserved, tightly regulated, reversible pathway during which ubiquitin, a small 76-amino acid, is transferred onto a target substrate facilitated by ubiquitin transferase and ligase enzymes [4]. The host enzymatic machinery involves a ubiquitin-activating enzyme E1, ubiquitin-conjugating enzyme E2, and a ubiquitin ligase enzyme E3 [4]. E1-activating enzymes initiate an ATP-dependent mechanism, resulting in a thioester bond between the active site of an E1-activating enzyme and the C-terminal Gly of ubiquitin [5]. Ubiquitin-conjugating E2 enzymes support the transfer of ubiquitin from the E1 enzyme onto the Cys residue of the E2 enzyme’s active site [6]. E3 enzymes continue supporting the ubiquitination pathway by orienting the target substrate and E2 enzyme to facilitate the transfer of ubiquitin to bind to the substrate. Humans have approximately 8 ubiquitin-activating E1 enzymes, 40 ubiquitin-conjugating E2 enzymes, and more than 600 ubiquitin-ligase E3 enzymes [7,8,9].

**Figure 1:**
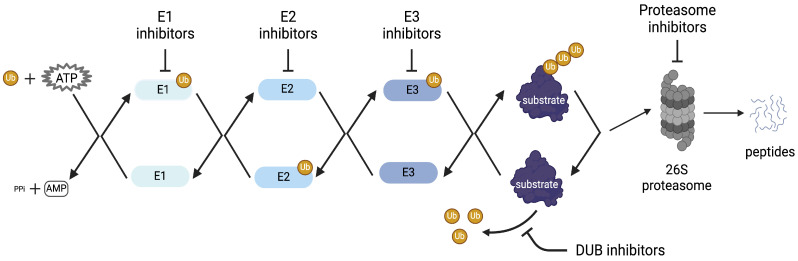
Schematic illustration of the potential small-molecule intervention points within the Ubiquitination Proteasomal System.

**Figure 2:**
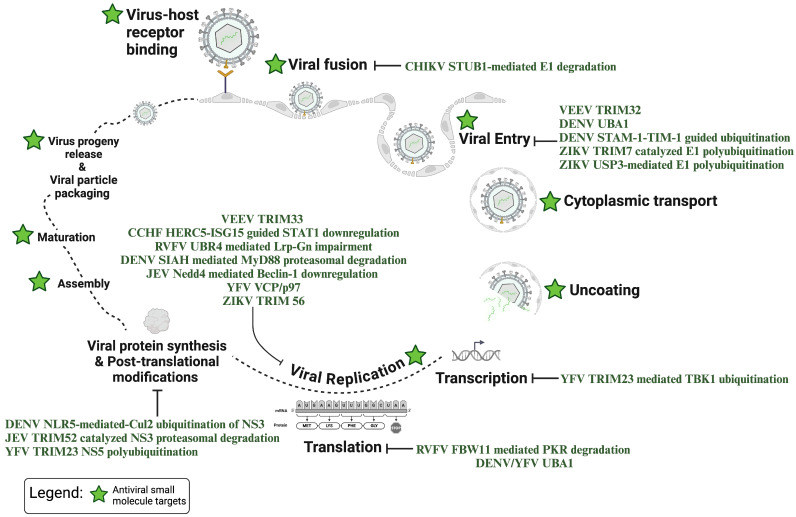
Illustration depicting stages of the virus life cycle targeted by ubiquitination pathways.

E3 ubiquitin ligases comprise a large family of proteins, playing a critical role in the transfer of ubiquitin to the target substrate of the ubiquitination pathway. E3 ligases are categorized into four groups: RING-finger E3 ligases, RBR E3 ligases (RING-IBR-RING), U-box E3 ligases, and HECT E3 ligases [10]. E3 ligases selectively recognize and control ubiquitylation of protein substrates, and account for substrate specificity observed in the ubiquitination pathway [11]. Degrons located within proteins determine the rate of degradation and serve as recognition points for E3 ligases [11]. Dysfunction of E3 ligases is known to impact human health and lead to several neurological diseases including Parkinson’s and Alzheimer’s disease [7]. E3 ligases also function as host proteins often hijacked by acutely infectious viral pathogens to facilitate the modification of viral proteins. Several E3 ligases are shared by many viral pathogens and play important roles in the viral replication cycle, thus laying the foundation for broad-spectrum therapeutic options (Figure 1 and Figure 2). The integral involvement of E3 ligases in regular host events also emphasizes the importance of closely monitoring toxicity and reversibility of function in the process of countermeasure development. In this review, we focus on three groups of mosquito-transmitted viral pathogens, namely alphaviruses, bunyaviruses, and flaviviruses, and the integral role of host machinery-mediated ubiquitination in the productive infectious process.

## 2. Alphaviruses

### 2.1. Chikungunya (CHIKV)

Chikungunya virus is an Old World (OW) alphavirus belonging to the *Togaviridae* family and is horizontally transmitted from *Aedes aegypti* and *Aedes albopictus* of the *Aedes* genus of mosquitoes to humans [12]. CHIKV is an enveloped, positive-sense, single-stranded RNA virus that sustains itself through sylvatic transmission cycles with humans as the primary reservoir [13,14]. Functioning as the causative agent of Chikungunya fever (CHIKVF), CHIKV is transmitted to vectors through blood meals of viremic hosts [15]. CHIKV was first identified in 1953 in an outbreak in Tanzania, Africa, and later isolated in the Makonde Plateau, Tanzania in 1952. Affecting more than one billion people, CHIKV can progress towards complex neurological manifestations of Guillain–Barre syndrome (GBS), meningoencephalitis, hepatitis, and myocarditis [16,17]. Surpassing 2.5 million cases, 35,000 CHIKV-related deaths targeting the elderly have been reported in the Americas and have resulted in over 158,00 disability-adjusted lifestyles [18,19]. Ixchiq (VLA1553), is the only single-dose FDA-approved CHIKV for humans, consisting of a live attenuated CHIKV strain [20].

Chikungunya virus is an Old World (OW) alphavirus belonging to the *Togaviridae* family and is horizontally transmitted from *Aedes aegypti* and *Aedes albopictus* of the *Aedes* genus of mosquitoes to humans [12]. CHIKV is an enveloped, positive-sense, single-stranded RNA virus that sustains itself through sylvatic transmission cycles with humans as the primary reservoir [13,14]. Functioning as the causative agent of Chikungunya fever (CHIKVF), CHIKV is transmitted to vectors through blood meals of viremic hosts [15]. CHIKV was first identified in 1953 in an outbreak in Tanzania, Africa, and later isolated in the Makonde Plateau, Tanzania in 1952. Affecting more than one billion people, CHIKV can progress towards complex neurological manifestations of Guillain–Barre syndrome (GBS), meningoencephalitis, hepatitis, and myocarditis [16,17]. Surpassing 2.5 million cases, 35,000 CHIKV-related deaths targeting the elderly have been reported in the Americas and have resulted in over 158,00 disability-adjusted lifestyles [18,19]. Ixchiq (VLA1553), is the only single-dose FDA-approved CHIKV for humans, consisting of a live attenuated CHIKV strain [20].

OW Alphaviruses are known to mediate shut-off host transcription and translation through Nsp2-regulated pathways [21]. OW Alphaviruses such as CHIKV utilize NsP2-mediated proteasomal degradation of Rpb1 to inhibit host transcription genes [22]. Although there is limited knowledge of NsP2-mediated translation, it is still a probable proviral mechanism initiated by OW Alphaviruses.

A study by Yin et al. reported a novel protein interaction between the viral CHIKV E1 protein and STIP1 homology and U-box-containing protein 1 (STUB1). This interaction exhibited STUB1’s, a known CHIKV host dependency factor, role in the ubiquitination and subsequent proteasomal degradation of E1 to promote viral replication [23]. Confirming STUB1’s role in the downregulation of CHIKV E1, the targeted siRNA-based silencing of STUB1 increased CHIKV E1 levels in 293T cells [23]. Further characterization of this interaction using GFP-trap-based immunoprecipitation revealed the K48-linked ubiquitination and degradation of CHIKV E1 by STUB1 [23].

### 2.2. New World Alphaviruses

Classified as New World (NW) Alphaviruses, Venezuelan Equine Encephalitis Virus (VEEV), Eastern Equine Encephalitis Virus (EEEV), and Western Equine Encephalitis Virus (WEEV) are positive-stranded RNA viruses under the family *Togavirdae* and genus *Alphavirus* [24]. These equine encephalitis viruses pose a significant threat in the Americas due to the associated short-term and long-term neurovirulent symptoms during infection [25]. VEEV, EEEV, and WEEV currently do not have any FDA-approved vaccine for humans, and treatment is based on preventive and supportive measures. From febrile flu-like symptoms to encephalitis, NW Alphaviruses are known to lead to encephalitis and mortality in humans [24,26]. NW Alphaviruses are arthropod-borne viruses vectored by a range of mosquito species: *Culex tarsalis*, *Culiseta*, and *Aedes* [27,28,29].

The Ubiquitin Proteasomal System (UPS) is important for the establishment of a productive VEEV infection, as determined by the FDA-approved small-molecule inhibitor, Bortezomib [30]. Using the live attenuated strain of VEEV (TC-83), it was shown that capsid was mono and polyubiquitinated and targeted for degradation during early infection [31]. This study demonstrated that UPS may support capsid degradation and promote the release of viral RNA in host-infected cells [31].

Host transcription shut-off is a virus-induced proviral mechanism mediated by capsid protein demonstrated by New World Alphaviruses after infection to inhibit interferon production and restrict antiviral gene expression [32,33]. NW alphavirus capsid protein forms complexes with export and nuclear factors to restrict nucleocytoplasmic transport and inhibit host transcription [22,32]. Based on NW alphaviruses’ inhibition of host transcription through capsid, a model in which VEEV TC-83 inhibits host mRNA transcription through K48-linked ubiquitination of the viral capsid protein has been suggested [31,32,34].

Notably, VEEV upregulates reactive oxygen species (ROS) to inflict host-based mitochondrial dysfunction and increase oxidative stress [35]. It has also been established that increased oxidative stress damages UPS activity and downregulates protein homeostasis resulting in aggregation of ubiquitin conjugates that may serve as oxidative stress indicators [36]. Hence, host-based oxidative stress inflicted by infection may impair UPS activity, function as a probable proviral mechanism in VEEV-TC-83, and extend to other Alphaviruses [35,36].

Implicated in functioning as proviral and antiviral restriction factors in flaviviruses, members of the TRIM E3 ligase family also play a role in alphavirus infection [37]. In VEEV TC-83 infected HeLa cells, TRIM33 operates as a proviral factor, while TRIM32 was established as an antiviral restriction factor [37]. TRIM33 increased VEEV-TC83-GRP infectivity, suggesting that TRIM33 is a target for antiviral small molecules [37]. TRIM32 is implicated in restricting the late entry step of VEEV before viral genome translation, in STING-mediated interferon production and the TBK1-independent pathway [37]. Additionally, VEEV-TC83-Nluc/Cap studies showed decreased activity in the presence of TRIM32 expression during the early stages of infection, suggesting that TRIM32 affects late VEEV entry by capsid uncoating [37].

## 3. Bunyaviruses

### 3.1. Crimean–Congo Hemorrhagic Fever (CCHF)

Crimean–Congo Hemorrhagic Fever Virus (CCHFV) is an enveloped, negative-sense RNA virus of the *Nairovidirae* family, *Bunyaviruales* order, transmitted either by exposure to infected blood or livestock, or through the bite of the infected *Hyalomma* tick, the main arthropod vector [38,39]. CCHFV, a hemorrhagic fever-causing virus, can proliferate through horizontal or vertical transmission cycles between ixodid ticks functioning as vectors and hosts, and amplifying in vertebrate hosts including cattle, ostriches, hares, goats, and sheep [30]. First identified in 1944 in Crimea, Ukraine, CCHFV is known to manifest through symptoms classified under four stages: incubation, pre-hemorrhagic, hemorrhagic, and convalescence stages. Despite being a highly prevalent tick-borne infectious disease with a fatality rate of 30%, CCHFV still has no FDA-approved vaccines available [38]. With CCHFV being a highly infectious virus and easily transmissible, finding a safe, effective treatment and the development of a vaccine to provide increased immunity remains a priority to protect those at risk of exposure.

CCHFV encodes a ubiquitin-specific protease belonging to the Ovarian Tumor Superfamily (OTU), functioning as a proviral mechanism as its deubiquitination activity is crucial for the production of particles [40]. Upon further study, the inactivation of the CCHFV OTU domain impeded CCHFV’s production of virions and reduced infectivity, indicating an active CCHFV OTU is needed for viral replication in cells [40]. The OTU domain-containing CCHFV protease codes for deISGylase and deubiquitase activity, functioning to reverse the ubiquitination of proteins and antagonizing interferon (IFN) production [40,41]. The OTU domain has two distinct functions, its deubiquitinase (DUB) and deISGylase activity, both specifically targeting ubiquitin-like interferon-stimulated gene 15 (ISG15) [40]. HECT and RCC1-CONTAING PROTEIN 5 (HERC5) is a HERC family E3 ubiquitin ligase responsible for an isopeptide bond between the c-terminal of ISG15 and the lysine of viral protein targets [40]. The HERC5 and ISG15 pathways are established regulators of antiviral immune response among viral infections including SARS-CoV-2 and HIV and may expand to CCHFV as well [40]. An interruption to the conjugation of ubiquitin and ISG15, affects IFN signaling, NFκB or signal transducer and activator of transcription 1 (STAT1), and other genes essential for the rapid response against viral infection [40,42]. Interfering with the OTU domain may provide key information into the potential development of a vaccine against CCHFV, but further research is required to find effective therapeutics and possible drug targets [42].

### 3.2. Rift Valley Fever Virus (RVFV)

Rift Valley Fever Virus, the causative agent of Rift Valley Fever (RVF), is an enveloped, negative-sense, mosquito-transmitted RNA virus belonging to the order *Bunyavirales* and the family *Phenuiviridae* [43]. RVF is an acute hemorrhagic illness primarily vectored by Culex and Aedes mosquito species [43,44]. Aedes mosquitoes serve as highly efficient primary vectors supporting the initial transmission of RVFV, while Culex mosquitoes serve as secondary vectors and aid in the maintenance of RVFV among mosquito populations [45]. Since its first discovery in 1931 of a sheep epidemic in the Rift Valley of Kenya, rural ruminants, newborn goats, and sheep have become highly susceptible to infection, characterized by high morbidity and 90–100% abortion rates [46,47]. With RVF being a zoonotic disease, viral transmission to humans is accomplished through contact with infected livestock and blood, body fluids, and mosquito bites resulting in a relatively low fatality rate [48]. Despite RVFV being classified as one of the eight pathogens on the WHO R&D blueprint list prioritized for further research and development, only inactivated and live-attenuated RVFV veterinary vaccines such as the MP12 strain and Rift Valley Fever Smithburn vaccine are available with no FDA-approved vaccines existing for human use [43,49,50]. RVFV is an overlooked re-emerging arbovirus with a steadily increasing disease burden in humans, strongly indicating the need for effective therapeutic intervention strategies apart from vector control alone.

Nonstructural S (NSs) protein is a key virulence factor for RVF, as it is implicated in type 1 interferon (IFN) antiviral response suppression mediated by the inhibition of host cell transcription and degradation of antiviral IFN effector [51]. The SCF E3 ligase complex comprises F-box proteins, Cullin-1, and Skp1, and is recruited and implicated in the proviral mechanisms initiated by RVFV [51]. RVFV NSs recruit E3 ligase F-box protein FBXO3 and E3 ligase Skp1 to degrade subunit p62 of host transcription general factor TFIIH and suppress IFN signaling [52]. As confirmed through siRNA-based knockdown studies, the depletion of FBX03 increased p62 TFIIH and IFN levels to demonstrate its proviral function in RVFV [53]. TFIIH is an important RNA polymerase 1 transcription factor and a common target for viruses to transcribe proviral genes, increase viral loads, and induce shut-off of host transcription [52,54]. Another proviral F-box protein, FBXW11, is also recruited by NSs to promote the degradation of the Protein Kinase R (PKR) pathway. PKR is a principal host antiviral defense mechanism as it inhibits the translation of viral mRNA to restrict RVF replication [55].

Distinct from the SCF complex, Ubiquitin Ligase E3 Component N-Recognin 4 UBR4, belonging to the HECT family, facilitates RVFV budding from the plasma membrane and viral protein transport to function as a proviral factor to promote RVF replication [55]. Limited knowledge exists on the interaction between Lrp, a crucial host factor for RVF pathogenesis, and Gn, but it is suggested that Gn is a glycoprotein precursor binding to host entry factors such as Lrp1 of RVF, aiding viral replication [56]. UBR4 functioning as an RVF Gn interactor may be implicated in the interruption of the interaction between Lrp1 and Gn to increase vRNA [55]. Depletion of UBR4 reduced RVF RNA and Gn expression levels, suggesting that UBR4 may increase RVF RNA levels and Gn interaction with host entry factor Lrp1 [55,56].

## 4. Flaviviruses

### 4.1. Dengue (DENV)

Dengue Virus (DENV) is an acutely infectious RNA virus transmitted by *Aedes* mosquitoes belonging to the family *Flaviviridae*, genus *Flavivirus* [57]. DENV is categorized into four distinct serotypes: DENV-1, DENV-2, DENV-3, and DENV-4. The first two serotypes of Dengue were isolated in Japan in 1943 (DENV-1) and Hawaii in 1945 (DENV-2) [58]. With more than 22,000 deaths and 400 million cases, all four Dengue serotypes have a combined annual death rate of 0.44% [59]. Notable short-term clinical symptoms associated with primary infection include high fever, headaches, vomiting, joint pain, and diarrhea [57]. Those who experience DHF eventually experience Dengue shock syndrome (DSS) and go through three distinct phases: febrile, critical, and convalescent [60]. Currently, Dengue has an FDA-approved vaccine Dengvaxia targeting all DENV serotypes alongside symptomatic-based treatments [61]. However, with the global disease burden associated with DENV infections, access and affordability of the tetravalent vaccine in resource-deprived settings, and challenges around protection in the context of heterotypic DENV infections, the need for robust broad-spectrum therapeutic strategies continue to be a critical unmet need. 

Ubiquitination independent of proteasomal activity is crucial to the uncoating of the DENV viral genome from endosomes and nucleocapsids [62]. Genome release and associated viral protein uncoating is a crucial step to all Flavivirus life cycles, including DENV infection, yet is poorly understood from the perspective of host–pathogen protein interaction partners and the specific modification requirements [62]. The inhibition of ubiquitin-activating enzyme 1 (UBA1), an E1-activating enzyme, through the small molecule PYR-41, deterred initial rounds of viral translation and reduced genome release during viral entry, verifying ubiquitination’s role in DENV pre-translation stage through nucleocapsid disassembly [62]. PYR-41 also confirms the necessity of non-degradable steps of ubiquitination to support uncoating, as DENV virions are trapped within endosomes and nucleocapsids restricting the DENV life cycle [63].

T-cell immunoglobulin and mucin domain 1 (TIM-1) is a phosphatidylserine host receptor and DENV entry receptor implicated in virus internalization [64]. The ubiquitination of TIM-1 is crucial to DENV endocytosis and the subsequent release of viral RNA into the host cells [63]. TIM-1 interacts with Signal transducing adaptor molecule 1 (STAM-1), a mediator of endocytosis of ubiquitinated cargo to facilitate viral entry and attachment to promote viral pathogenesis [64].

Additionally supporting the ubiquitination pathway, and the role of E3 ligases in antiviral intervention, the ubiquitination of an E3 ligase, Cullin-2 (Cul2) also impacts DENV infection [65]. Nod-like receptor (NLR) family card domain containing 5 (NLRC5), a protein implicated in innate immunity, acts as an adaptor to recruit Cul2 to catalyze K48-linked polyubiquitination of nonstructural viral protein 3 (NS3) to significantly suppress DENV infection [65].

SIAH1 is another E3 ligase that also plays a crucial role in suppressing MyD88 in DENV2-infected cells to promote viral replication, thus alluding to the downregulation of host antiviral responses by ubiquitination [66]. MyD88 is an antiviral host protein that mediates NF-KB signaling, ubiquitinated and suppressed by SIAHI in DENV2. Knockdown of SIAHI led to proteasome-dependent degradation of MyD88 and restricted DENV2 infection [66].

### 4.2. Japanese Encephalitis Virus (JEV)

Japanese Encephalitis Virus (JEV), the causative agent of Japanese encephalitis (JE), is a positive-sense, enveloped, RNA virus belonging to the *Flaviviridae* family, and genus *Flavivirus* [67]. JEV is an arbovirus vectored by Culex mosquito species, maintained in an intricate transmission cycle by circulating among various amplifying and reservoir vertebrate hosts [68]. Members of the *Suidae* and the avian *Aviradae* family are established as prominent amplifying hosts, adept at becoming viremic and aiding the transmission of JEV [68]. Water birds and potentially insectivorous bats function as natural reservoirs of JEV, while equine and human populations are considered incidental hosts with limited onward transmission capacity [68,69]. With JEVs’ history of intercontinental expansion since its isolation in 1935, its varied range of hosts exacerbates JEVs’ threat to public health with more than 60,000 cases annually worldwide [70,71]. JEV has only one serotype but has five distinct genotypes identified: I, II, III, IV, and V [72]. JEV includes clinical symptoms of nausea, vomiting, headache, and fever with 1% of infected patients progressing to encephalitis, with 20–30% mortality [73]. Despite the highly efficacious IXARO vaccine available, JEV infections are still common globally, underscoring the necessity of post-infection therapeutic intervention strategies [74,75]. Understanding the role ubiquitination plays in the host in terms of immune response and antiviral defense in JEV and the virus, to aid in the establishment of a productive infection, will lay the groundwork for an effective post-infection treatment strategy.

Ubiquitination plays a prominent role in the host–virus interactions that support viral entry of JEV [76]. In JEV, neural precursor cell-expressed, developmentally down-regulated 4 (Nedd4) and tripartite-motif-containing protein (TRIM)-regulated ubiquitination pathways hold insight into host antiviral defense mechanisms [77,78]. The TRIM family is the largest group of RING-finger E3 ubiquitin ligases with members regulating innate immune responses in viral infection to function as proviral and host restriction factors [79,80]. TRIM21 functions as a proviral factor to increase JEV replication, while TRIM52 functions as a host restriction factor to reduce JEV replication [77]. TRIM21, a known regulator of interferon beta (IFN-β), downregulated IFN-β in infected human microglial cells [77]. TRIM52 functions as a host restriction factor and degrades viral nonstructural protein 2A (NS2A) of JEV by targeting it for proteasomal degradation, and the restriction of viral replication [77,81].

Beyond the TRIM family, Nedd4 also functions as a proviral factor to upregulate the JEV replication [78]. Nedd4 is an E3 ligase, belonging to the HECT family, not implicated in the viral entry of JEV, but is upregulated in SK-N-SH neuroblastoma cells infected with JEV [78]. Nedd4 knockdown studies with RNA interference resulted in a significant reduction in JEV viral load and JEV NS3 expression [78]. Nedd4 suppressed viral-induced autophagy through the downregulation of autophagy and ubiquitination mediator Beclin-1 to increase JEV viral load [78].

### 4.3. Yellow Fever Virus (YFV)

Yellow Fever Virus (YFV), is another RNA virus of the *Flaviviridae* family, genus *Flavivirus* with prominence in low-income countries [82]. Despite an effective YF vaccine available to the public for 80 years, YF is still endemic due to low vaccination coverage in Africa and South America [82,83]. YFV, a single-stranded, positive-sense, arbovirus is transmitted through infected *Aedes* and *Haemagogus* species mosquitoes and is known to cause acute viral hemorrhagic disease [82]. YFV establishes itself among hosts through sylvatic, intermediate, and urban secondary transmission cycles [84]. Transmitted through wild and urban environments, this acute febrile disease results in 200,000 cases annually, with 30,000 cases resulting in death worldwide [85]. Based on the live attenuated 17D YFV strain, YF-VAX, and Stamarail, FDA-approved vaccines are available in the United States [86]. Even with 17D hailed as a gold standard in live attenuated vaccines attributable to its high seroconversion rates, YF vaccine-associated neurotropic disease (YFL-AND) complications have been observed following YF vaccinations. Given the adverse manifestations observed in vulnerable groups, and the growing disease burden in areas where the mosquito vectors are endemic, having a post-infection treatment strategy is imperative.

Similar to ubiquitination’s role in the viral internalization of other flaviviruses, the YFV genome uncoating is also ubiquitination dependent. Demonstrated by a YFV entry luciferase reporter virus, YFVΔSK/Nluc, Valosin-containing protein (VCP/p97) dissembles nucleocapsids during post-fusion and pre-translational steps in YFV entry [87]. Validated by VCP/p97 inhibitors, VCP/p97 is a host protein aiding the initial replication of RNA viruses [88]. Confirmed by the inhibition of ubiquitination through the E1 inhibitor PYR-41, UBA1-mediated ubiquitination was deemed to be essential to the initial rounds of YFV translation with the proposition that ubiquitin may tag nucleocapsids for uncoating by host factors [87].

With a limited understanding of ubiquitination during YFV replication, the role played by E3 ligases in YFV replication is even less understood. TRIM23 was observed to be important to antiviral innate immune responses as it initiates TANK-binding kinase 1 (TBK1) to target viral proteins through autophagy [89]. However, YFV among other flaviviruses can hijack and target previously established host antiviral E3 ligases, such as TRIM23, to promote viral replication. TRIM23 functions as a proviral factor in YFV replication as it polyubiquitinates YFV nonstructural NS5 proteins to increase signal transducer and activator of transcription 2 (STAT2) binding and inhibit antiviral type 1 interferon signaling [89].

### 4.4. Zika Virus (ZIKV)

Among the many emerging and re-emerging viral pathogens, Zika virus (ZIKV) poses a severe public health concern [90]. ZIKV is a positive-sense, single-stranded RNA virus primarily vectored by the *Aedes aegypti* mosquito [90,91]. Since its initial identification in 1947 Zika Forest, Uganda from a rhesus monkey, this neurotropic flavivirus has diverged into two distinct Asian and African lineages [92,93]. The African lineage of ZIKV has a sylvatic transmission cycle between nonhuman primates such as apes and monkeys and humans and *Aedes* mosquitoes as hosts [94]. The Asian lineage of ZIKV is prominently studied and circulates through an urban cycle with transmission occurring between humans and *Aedes* mosquitoes [95]. ZIKV displays three modes of transmission: horizontal, vertical, and venereal [95]. ZIKV has also been noted to be sexually transmitted, and vertically transmitted from mother to fetus, with vRNA detected in saliva, blood, amniotic fluid, and breast milk [90]. ZIKV is still a major concern among flaviviruses, as it has consequential neurological manifestations alongside the development of acute Guillain–Barre Syndrome in adults and microcephaly in newborns [90,96]. With most ZIKV-related vaccine studies cemented in preclinical or phase 1 clinical studies and no FDA-approved therapeutic intervention strategies, the vital need to explore antiviral targets as part of host-based machinery is emphasized [97].

Like DENV, ubiquitination is also advantageous in ZIKV pathogenesis as it supports viral attachment and entry. Among the 3 structural ZIKV virion proteins, the structural Envelope (E) protein is K63-linked polyubiquitinated by E3 ligases TRIM 7 to enhance viral replication [98]. The ZIKV E protein is implicated in the facilitation of receptor binding during viral entry and mediates virus-endosome membrane fusion after attachment [99]. TRIM7 knockdown mouse models confirmed TRIM7 downregulated viral replication, indicating the TRIM7-mediated ubiquitination of ZIKV E is a proviral pathway in ZIKV [98]. The overexpression of another TRIM E3 ligase, TRIM56 in ZIKV-infected cell lines inhibits ZIKV viral replication, supported by TRIM56 depletion studies resulting in elevated vRNA levels [100]. With TRIM56 functioning as an RNA-binding protein, the TRIM56-ZIKV pathway suggests antiviral properties.

Additionally, Ubiquitin-Specific Peptidase 38 (USP38)-mediated ubiquitination inhibition of ZIKV replication, also confirms ubiquitination’s role in virus internalization [101]. USP38 is a deubiquitinating enzyme, belonging to the USPS family that reverses ubiquitination through the removal of ubiquitin from target proteins [101]. USP38 binds to the C-terminal domain of ZIKV E protein to impair K63-linked polyubiquitination resulting in the inhibition of ZIKV infection [101]. 

## 5. Expert Opinion

### 5.1. Small Molecules Targeting Vector Transmitted RNA Viruses Ubiquitin Pathways

Due to the importance of ubiquitination during the viral life cycle of these vector-transmitted RNA viruses, many studies have been conducted on developing and identifying small-molecule inhibitors to target ubiquitin-related processes. It is well known that the ubiquitin-proteosome (UPS) system plays an important role in the life cycle of a multitude of viruses and that the inhibition of such processes can lead to significant inhibition of viral attachment and replication (Table 1) [102,103]. In this regard, a small-molecule proteasome inhibitor known as IU1 has been shown to significantly decrease viral replication of multiple flaviviruses, with robust inhibition seen with DENV [104]. IU1 inhibits the proteosome-associated deubiquitinating enzyme USP14, which belongs to a large family of deubiquitinating enzymes (DUBs). High-resolution co-crystal structures of USP14 and IU1 have shown inhibition occurs through an allosteric regulation of USP14 by IU1 that inhibits the binding of ubiquitin to the active site of USP14 [105]. Because ubiquitination is a reversible process, DUBs play important roles in maintaining a balance within the UPS system.

Ubiquitination has also been heavily indicated to play a significant role in the endoplasmic reticulum-associated protein degradation (ERAD) pathway [107]. Multiple CRISPR-Cas9 and RNAi screens have revealed several Hrd1-mediated ERAD pathway-associated regulatory host factors crucial for flavivirus replication [108,109,110,111,112]. Hrd1 (Hydroxymethyl glutaryl-coenzyme A reductase degradation protein 1) is an ER-associated transmembrane E3 ubiquitin ligase that plays an important role in the ERAD-mediated degradation of misfolded proteins [113]. It was hypothesized that because multiple Hrd1-mediated ERAD pathway host factors were identified to be crucial for flavivirus replication, an inhibitor of the Hrd1 complex that plays a role in the dislocation of ubiquitinated proteins from the ER to the cytosol may exhibit antiviral activity against multiple flaviviruses [114]. As such, a screen of small molecules determined that a dislocation inhibitor compound 26 (CP26) exhibited potent anti-ZIKV and anti-DENV activity [114]. It was determined that CP26 enhances the thermal stability of select proteins within the Hrd1 complex, inhibits ubiquitination and degradation of misfolded ER proteins, and ultimately significantly inhibits the replication of both ZIKV and DENV in HUH-7 cells [114]. This inhibition highlights the potential for therapeutics specifically targeting ER dislocation and related E3 ligase activity. Further studies have been conducted to reveal small-molecule inhibitors of ER dislocation specifically focused on E3 ligase activity such as RNF5 [112]. In fact, in recent years, an intracellular ZIKV NS2AB3 self-cleavage assay identified the proteasome inhibitor bortezomib as a potent inhibitor of both ZIKV and DENV [115]. It was determined that bortezomib specifically promoted the aggregation and ubiquitination of NS3 and that the E3 ligases RNF126 and HRD1 were responsible for the associated ubiquitination; again highlighting the potential for targets against ERAD-associated processes and the importance of E3 ligases [115].

In a similar manner to the inhibition of ZIKV and DENV, the proteasomal inhibitor bortezomib has demonstrated potent inhibition of the alphaviruses VEEV, WEEV, and EEEV [31]. This inhibition is believed to be related to the early ubiquitination of K48 on the VEEV capsid that leads to ubiquitin-mediated degradation of the capsid and subsequent release of viral genomic RNA within the cytoplasm, which bortezomib interferes with [31]. In the alphavirus CHIKV, it has been demonstrated that DUBs inhibitors such as PR619 and WP1130 significantly reduce CHIKV replication in HEK293T, Vero-E6, and Huh-7 cells, in a mechanism that impairs viral RNA and protein synthesis [116]. Interestingly, it was shown in the same study that the expression levels of 6 DUBs (USP7, USP10, CYLD, UCHL1, STAMBP, otubain A20) were not regulated by the CHIKV infection, but A20 and UCHL1 were upregulated in measles, human papillomavirus (HPV), and influenza virus [116,117,118,119]. In RVFV infection, it has been again shown that bortezomib has potent inhibitory effects on viral replication in human cell lines [120]. In this case, bortezomib specifically affects the ubiquitination of SAP30 and mSin3a, which interact with the nonstructural S-segment protein (NSs) [120].

### 5.2. Small Molecules Targeting Other Virus Ubiquitin Pathways

The importance of small-molecule targets against ubiquitination-related processes as therapeutic agents extends further than just vector-transmitted RNA viruses (Table 2). For example, the Infected cell protein 0 (ICP0) of herpes simplex virus 1 (HSV-1) plays an important role as an immediate early gene that inhibits the host antiviral response as well as allows for early viral gene expression, which is believed to occur by a mechanism that induces the degradation of targeted proteins through E3 ubiquitin ligase activity [121]. As such, a high-throughput assay was developed based on the autoubiquitination of ICP0 to screen for potential small molecules that inhibit the E3 ubiquitin ligase activity of ICP0 [121]. The resulting screen identified a 3,4,5-aryl-substituted isoxazole small molecule that exhibited significant HSV-1 inhibition in an ICP0-dependent manner which is thought to occur by inhibiting the E3 ligase function of ICP0 [121]. In the Sendai virus, vesicular stomatitis virus, and influenza A virus, it has been shown that viral infection promotes the expression of the deubiquitinase ubiquitin-specific protein 7 (USP7) which negatively regulates the IFN-I antiviral response [122]. However, treatment with the small-molecule inhibitors of USP7, P5091, and P22077 leads to enhancement of the IFN-1 antiviral response in vitro [122]. This process is believed to occur by destabilizing the suppressor of cytokine signaling 1 protein (SOCS1) that USP7 enhances stability through deubiquitination [122]. Similarly, in both human Norwalk and murine noroviruses, the small-molecule DUBs inhibitor WP1130 has been shown to decrease norovirus replication [123]. Further examination revealed that WP1130 targets the DUB USP14, which binds to inositol-requiring enzyme 1 (IRE1), indicating again that the unfolded protein response (UPR) pathway plays an important role during viral infection [123]. In HIV-1, it has been shown that apolipoprotein B mRNA-editing catalytic polypeptide-like 3 family members (APOBEC3) plays an important role as a host antiviral factor that HIV-1 viral infectivity factor (Vif) targets for proteasomal degradation through an E3 ubiquitin ligase-dependent manner facilitated by the core-binding factor β protein (CBFβ) [124,125,126,127,128,129,130]. In recent years however, it has been shown that the inhibitor CV-3 significantly inhibits HIV-1 replication, recovers APOBEC3 activity, and specifically inhibits the interaction of CBFβ and Vif [130], again highlighting the potential of small-molecule inhibitors of ubiquitin-related processes as potential therapeutics.

## 6. Conclusions

The dependence of protein interactions involving viral and host protein partners on host-mediated enzymatic processes in the context of these vector-transmitted RNA viruses underscores how these host processes can deliver broadly effective therapeutic intervention strategies (Table 3). Notably, many small-molecule inhibitors that impact the E1, E2, and E3 enzymes, and the downstream proteasome are FDA approved for alternate indications, thus offering opportunities for drug repurposing. Often, it is noted that effective concentrations of these host-based enzyme inhibitors are in the low micromolar to nanomolar range, thus alluding to attainable safety profiles when delivered in a therapeutic capacity. With further expansion of the knowledge base around the impact of ubiquitination on specific viral life cycle events such as entry, uncoating, RNA synthesis, assembly, and egress, it may also be possible to narrow the treatment window to further limit potential safety concerns. While we have focused exclusively on the ubiquitination enzymatic machinery in this review, ubiquitin-mediated host signaling events that contribute to several highly conserved cellular innate immune signaling mechanisms provide excellent opportunities to further expand the target portfolio, towards delivering broad-spectrum, host-based therapeutic solutions against these vector-transmitted viral pathogens.

## Figures and Tables

**Table 1:** A comprehensive table of E3 ligase-mediated ubiquitin pathways specific to viruses mentioned in the review.

Virus	Host or Viral Protein	Target Ub Pathway	Mechanism of Action	Proviral or Antiviral	Reference
CHIKV	Host/Viral	STUB1-mediated E1 degradation	Viral Fusion	Antiviral	[23]
VEEV	Host	TRIM33	Viral Replication	Proviral	[37]
	Host	TRIM32	Late Viral Entry	Antiviral	[37]
CCHF	Host	HERC5-ISG15-guided STAT1 downregulation	Viral Replication	Proviral	[106]
RVFV	Host	Skp1 and FBXO3-mediated p62 TFIIH degradation	Host Transcription	Proviral	[52,53]
	Host	FBXW11-mediated PKR degradation	Viral Translation	Proviral	[55]
	Host	UBR4-mediated Lrp-Gn impairment	Viral Replication	Proviral	[55]
DENV	Host	UBA1	Viral Entry/Translation	Antiviral	[62]
	Host	STAM-1-TIM-1-guided ubiquitination	Viral Entry	Proviral	[63,64]
	Host	NLRC5-mediated-Cul2 K48-linked polyubiquitination of NS3	Viral Replication	Antiviral	[65]
	Host	SIAH-mediated MyD88 proteasomal degradation	Viral Replication	Proviral	[66]
JEV	Host	TRIM52 catalyzed NS3 proteasomal degradation	Viral Replication	Antiviral	[77]
	Host	Nedd4-mediated Beclin-1 downregulation	Viral Replication/NS3 Expression	Proviral	[78]
YFV	Host	TRIM23-mediated K63-linked polyubiquitination of NS5	Viral Replication	Proviral	[89]
	Host	TRIM23-mediated TBK1 ubiquitination	Viral Replication/Transcription	Antiviral	[89]
	Host	VCP/p97	Initial Viral Replication	Proviral	[88]
	Host	UBA1	Initial Viral Translation	Proviral	[87]
ZIKV	Host/Viral	TRIM7 catalyzed K63-linked E1 polyubiquitination	Viral Entry/Attachment	Proviral	[98]
	Host	USP3-mediated impairment of K63-linked ZIKV E1 polyubiquitination	Viral Entry	Antiviral	[101]
	Host	TRIM56	Viral Replication	Antiviral	[100]

**Table 2:** A comprehensive table mentioning proviral and antiviral small molecules targeting ubiquitination pathways specific to each virus in review.

Small Molecule	Function	Effect	Antiviral/ Proviral	Virus	Reference
**IU1**	Inhibits DUB USP14	Inhibits viral replication	Antiviral	DENV	[104]
**CP26**	Inhibits Hrd1- mediated ERAD pathway	Inhibits ubiquitination and degradation of misfolded ER proteins	Antiviral	DENV/ZIKV	[113]
**PYR-41**	Inhibits ubiquitin- activating enzyme E1 (UBA1)	Restricts viral translation	Antiviral	DENV	[131]
**Bortezomib**	Proteasomal inhibitor	Ubiquitin-mediated capsid degradation	Antiviral	VEEV	[31]
		Inhibits viral replication	Antiviral	VEEV/EEEV/WEEV	[31]
		NS3 ubiquitination	Antiviral	DENV/ZIKV	[115]
		SAP30 and mSin3A ubiquitination	Antiviral	RVFV	[120]
**PR619**	Broad-spectrum DUB inhibitor of USP and UCHL5 family.	Impairs viral RNA and protein synthesis	Antiviral	CHIKV	[116]
**WP1130**	DUB inhibitor of USP5, USP9X, USP14, USP37, and UCHL5	Impairs viral RNA and protein synthesis	Antiviral	CHIKV	[116]
**WP1130**	Targets DUB USP14	Binds to IRE1	Antiviral	Human Norwalk/Murine noroviruses (MNV)	[123]
**CV-3**	Rescues APOBEC3 activity and inhibits the interaction between Vif and CBFβ	Inhibits HIV-1 replication	Antiviral	HIV-1	[130]

**Table 3:** Comprehensive table of viral and host proteins, their proviral or antiviral nature, their mechanism of action, and the targeted stage of virus life cycle specific to each virus mentioned.

Virus	Protein	Viral/Host Protein	Proviral/ Antiviral	Mechanism of Action	Targeted Stage of Virus Life Cycle	Reference
CHIKV	NSP2	Viral	Proviral	Inhibits host transcription and translation	NA	[22]
	Rpb1	Host	Proviral	Inhibits antiviral host genes	NA	[22]
	E1	Viral	Proviral	Viral fusion	Viral fusion	[23]
	STUB1	Host	Proviral	CHIKV host dependency factor	Degrades CHIKV E1	[23]
	CYLD	Host	Antiviral	DUB	NA	[116]
	A20	Host	Antiviral	DUB	NA	[116]
	UCHL1	Host	Antiviral	DUB	NA	[132]
	STAMBP	Host	Antiviral	DUB	NA	[133]
	Otubain A20	Host	Antiviral	DUB	NA	[134]
	USP10	Host	Antiviral	DUB	NA	[135]
VEEV	Capsid	Viral	Proviral	Inhibits host transcription	Increases vRNA	[31]
	TRIM33	Host	Proviral	Unknown	Unknown	[37]
	TRIM32	Host	Antiviral	Capsid uncoating	Late viral entry	[37]
CCHF	ISG15	Host	Antiviral	Immune response regulator	NA	[106]
	IFN	Host	Antiviral	Antiviral response	NA	[41]
	HERC5	Host	Antiviral	Immune response regulator	NA	[106]
	NK-FB	Host	Antiviral	Immune response regulator	NA	[41,106]
	STAT1	Host	Antiviral	IFN response mediator	NA	[41,106]
RVFV	NSs	Viral	Proviral	RVFV virulence factor	Suppresses IFN signaling	[53]
	Cullin-1	Host	Proviral	Activates PKR	NA	[53]
	Skp1	Host	Proviral	Degrade p62 TFIIH	NA	[53]
	FBXO3	Host	Proviral	Degrade p62 TFIIH	NA	[53]
	FBXW11	Host	Proviral	PKR degradation	Viral translation	[55]
	TFIIH	Host	Antiviral	IFN signaling	NA	[53]
	UBR4	Host	Proviral	RVFV Gn interactor	Viral protein Transport	[55,56]
	Lrp	Host	Antiviral	RVFV host restriction factor	NA	[55,56]
	Gn	Host	Proviral	Binds to Lrp	NA	[55,56]
	PKR	Host	Antiviral	Inhibits viral translation	NA	[55]
	SAP30	Host	Proviral	NSs interactors, inhibit IFN-β expression	Unknown inhibition	[120]
	mSin3a	Host	Proviral	NSs interactors, inhibit—β expression	Unknown inhibition	[120]
DENV	TIM-1	Host	Proviral	DENV entry receptor	Viral endocytosis	[63,64]
	STAM-1	Host	Proviral	Ubiquitinated cargo mediation	Viral entry and attachment	[63,64]
	NS3	Viral	Proviral	RNA replication	RNA replication	[65]
	SIAH	Host	Proviral	Ubiquitinates, degrades MyD88	NA	[66]
	MyD88	Host	Antiviral	Mediates NF-KB signaling	Unknown inhibition	[66]
	Cullin-2	Host	Antiviral	Polyubiquitinates NS3	Unknown inhibition	[65]
	NLC5	Host	Antiviral	Innate immunity	Unknown inhibition	[65]
JEV	Nedd4	Host	Proviral	Downregulates Beclin-1	Increases NS3 viral protein	[77,78]
	Beclin-1	Host	Antiviral	Autophagy and ubiquitination regulator	Unknown inhibition	[77,78]
	TRIM21	Host	Proviral	Downregulates IFN-β signaling	Viral replication	[77]
	TRIM52	Host	Antiviral	Host restriction factor	Degrades viral NS2A	[81]
	NS2A	Viral	Proviral	Recruits vRNA	Viral replication	[81]
	NS3	Viral	Proviral	Protease activity	Viral replication	[78]
YFV	VCP/p97	Host	Proviral	Protein homeostasis	Post-fusion and pre-translation	[87,88]
	TRIM23	Host	Proviral	Initiates TBK1	Unknown inhibition	[89]
	TRIM23	Host	Antiviral	Inhibit IFN signaling	Polyubiquitinates NS5	[89]
	NS5	Viral	Antiviral	Interferon signaling mediator	Unknown inhibition	[89]
	STAT2	Host	Proviral	Inhibit type-1 IFN	Viral replication	[89,98]
ZIKV	E	Viral	Proviral	Receptor binding	Viral entry	[98,99]
	TRIM7	Host	Proviral	Virus endosome membrane fusion	Viral entry	[89]
	TRIM56	Host	Antiviral	RNA binding protein	Downregulates vRNA	[100]
	USP38	Host	Antiviral	Binds to ZIKV E	Inhibits viral Replication	[101]
	ZIKV NS2A3	Viral	Proviral	Cleaves ZIKV polyprotein precursor	Viral protein Generation	[136]
	RNF126	Host	Antiviral	Ubiquitination of ZIKV NS3	NA	[115]
	NS3	Viral	Proviral	Promotes NS5-guided RNA synthesis	Viral replication	[115]
DENV/ ZIKV	Hrd1	Host	Antiviral	ERAD-mediated degradation of misfolded proteins	NA	[113]
	Hrd1	Host	Antiviral	Ubiquitination of ZIKV NS3	NA	[115]
Norovirus	USP14	Host	Proviral	DUB binds to IRE1	Viral replication	[104]
Measles Influenza HPV	UCHL1	Host	Proviral	Downregulate immune response	Viral replication	[116]
	A20	Host	Proviral	TRAF6 ubiquitination modification	Viral replication	[116]
HIV-1	CBFB	Host	Proviral	Interacts with Vif-mediated cell cycle regulation	Unknown host inhibition	[137]
	APOBEC3	Host	Antiviral	Targeted for proteasomal degradation through CBFB	Unknown inhibition	[137]
	Vif	Viral	Proviral	HIV-1 accessory protein, degrades APOBEC3	Viral replication	[137]
HSV-1	SOCS1	Host	Proviral	Downregulation of IFN-1 expression	Viral replication	[122]
	USP7	Host	Proviral	Deubiquitinates and promotes SOCS1 stability	Viral immune evasion	[122]
